# *BRCA1* and *BRCA2* germline mutation spectrum and frequencies in Belgian breast/ovarian cancer families

**DOI:** 10.1038/sj.bjc.6601656

**Published:** 2004-02-24

**Authors:** K Claes, B Poppe, I Coene, A De Paepe, L Messiaen

**Affiliations:** 1 Centre for Medical Genetics, Ghent University Hospital, De Pintelaan 185, Gent 9000, Belgium; 2 Department of Genetics, University of Alabama, 1530 3rd Ave. S., Kaul 420, Birmingham, AL 35249-0024, USA

**Keywords:** hereditary breast cancer, *BRCA1*, *BRCA2*, mutation analysis, HBOC

## Abstract

Worldwide variation in the distribution of *BRCA1* and *BRCA2* mutations is well recognised, and for the Belgian population no comprehensive studies about *BRCA1/2* mutation spectra or frequencies have been published. We screened the complete coding region of both genes in 451 individuals from 349 Belgian families referred to a family cancer clinic and identified 49 families with a *BRCA1* and 26 families with a *BRCA2* mutation. Six major recurrent mutations (*BRCA1* IVS5+3A>G, 2478–2479insG, E1221X and *BRCA2* IVS6+1G>A, 6503-6504delTT, 9132delC) accounted for nearly 60% of all mutations identified. Besides 75 true pathogenic mutations, we identified several variants of unknown clinical significance. In combination with a family history, an early average age of female breast cancer diagnosis (*P*<0.001), and the presence of a relative with ovarian cancer (*P*<0.0001) or multiple primary breast cancers (*P*=0.002), increased the chance for finding a mutation. Male breast cancer was indicative of a *BRCA2* mutation segregating in the family (*P*=0.002). Mutations in the 5′-end of *BRCA1* and *BRCA2* were associated with a significantly increased risk for ovarian cancer relative to the central portion of the gene. Our study suggests a role for additional breast cancer susceptibility genes in the Belgian population, since mutation detection ratios were low in high-risk breast cancer-only families as compared to breast–ovarian cancer families. Given the large proportion of recurring mutations, molecular testing can now be organised in a more cost-effective way. Our data allow optimisation of genetic counselling and disease prevention in Belgian breast/ovarian cancer families.

Since the mapping and the cloning of two genes that confer susceptibility to both breast and ovarian cancer, *BRCA1* and *BRCA2* (Miki *et al*, 1994; Wooster *et al*, 1995; Tavtigian *et al*, 1996), it became possible to offer genetic testing to families with a predisposition for breast and/or ovarian cancer. Consequently, individuals at risk can now be identified as candidates for surveillance programmes. A large number of distinct mutations in the *BRCA1* and *BRCA2* genes have been reported worldwide, but population-specific variation in the distribution of *BRCA1/2* mutations is well recognised. In some populations or ethnic groups, founder mutations form a sufficient proportion of the total to justify the adoption of specific molecular screening strategies.

To date, no comprehensive studies in the Belgian population have been published. Only data from small series are available (Claes *et al*, 1999a, 1999b) or from studies in which the analysis was restricted to a few *BRCA1/2* exons or to *BRCA1* only (Peelen *et al*, 1997; Sibille-Hoang *et al*, 1998; Goelen *et al*, 1999). We performed mutation screening of the complete coding region of *BRCA1* and *BRCA2* in 349 unrelated Belgian families referred to our family cancer clinic and report here the nature and distribution of the mutations identified. We found phenotypic differences between families in whom a disease-causing mutation was identified *vs BRCA1/*2 mutation-negative families. We also evaluated in our cohort of patients if we could find an association between mutation site and relative risk of breast or ovarian cancer.

## MATERIALS AND METHODS

### Study population

Breast and/or ovarian cancer-prone families visiting the Centre for Medical Genetics at the Ghent University Hospital were selected for molecular testing of the *BRCA1/2* genes if fulfilling one of the following inclusion criteria:

1. families with at least three first-degree relatives(^*^) with breast and/or ovarian cancer;
2. families with at least two first- and/or second-degree relatives(^*^) with breast and/or ovarian cancer before an average age of 50 years;
3. sporadic patients diagnosed with breast or ovarian cancer before the age of 38 years;
4. sporadic patients diagnosed with multiple primary breast cancers or concomitant breast and ovarian cancer and all tumours occurred before an average age of 50 years; and
5. sporadic patients with male breast cancer

(^*^) in case of male gene transmission, two affected females related through a male were considered to be first-degree relatives.

Counselling and genetic testing were provided by a multidisciplinary team of genetic counsellors, gynaecologists, oncologists, a psychologist and molecular biologists (De Vos *et al*, 1999). Before taking a blood sample, an informed consent was obtained. This study was approved by the ethics committee of the Ghent University Hospital.

In total, 58 sporadic patients (without a family history) and 291 families with a history of breast and/or ovarian cancer were analysed. To investigate familial clustering of the disease, we made a distinction between families with hereditary breast and/or ovarian cancer (HBOC) and familial breast and/or ovarian cancer (FBOC). Hereditary breast and/or ovarian cancer families are at high risk (>3 times population risk) and defined as families with at least three first-degree relatives with breast and/or ovarian cancer (or second-degree relatives in case of paternal inheritance) in at least two successive generations and at least one of them diagnosed before the age of 50 years. Families with at least two first-degree relatives (or second-degree relatives, in case of paternal inheritance) with breast and/or ovarian cancer diagnosed at young age, but not fulfilling the criteria for HBOC, are FBOC families. These families are at moderate increased risk (two to three times population risk). In total, we analysed 91 HBOC families and 200 FBOC families (Table 1

**Table 1:** Unclassified variants in *BRCA1* and *BRCA2*

**Exon/intron**	**Nucleotide change**	**Amino-acid change**	**BIC**	**No. of families**	**Type of family**	**Polarity change**	**Conserved in dog/mouse/rat/chicken**	**Pathogenic status**
*BRCA1*								
2	IVS2–14C>T	Noncoding	Y	1	F, brca only	/	/	UV/P (no aberrant transcript observed by cDNA analysis) Claes *et al* (2003)
10	IVS10+8C>T	Noncoding	N	1	Sporadic	/	/	UV/P (no aberrant transcript observed by cDNA analysis)
11	2196G>A	D693N	Y	3	F, brca-only^a^ F, brovca; H, brca only	N (negatively charged → uncharged polar)	N/N/N/N	UV/P (in two of the families true pathogenic *BRCA2* mutations (IVS6+1G>A, 1617–1618delAG) were segregating)
11	3238G>A	S1040N	Y	3	H, brca-only^a^ F, brovca; F, brca only	n	N/Y/Y/N	UV/P [in two of the families pathogenic mutations were segregating (*BRCA1* E1221X, *BRCA2* 1617–618delAG)]
11	3298A>C	E1060A	Y	5	Sporadic^a^ F, brca-only; F, brovc^a^ H, brca only	N (negatively charged → positively charged)	Y/Y/Y/N	UV/P (variant in linkage disequilibrium with *BRCA1* E1221X)
11^b^	4145A>C	S1342S	N	1	H, brca only	/	/	UV
17	5112G>A	V1665M	Y	1	H, brca only	N	Y/Y/Y/Y	UV/P (not segregating with the disease)

*BRCA2*								
3	407A>G	N60S	Y	1	F, brca only	N	Y/N/N/N	UV
4	IVS4+33A>G	Noncoding	N	1	F, brovca	/	/	UV/P (patient is carrying a pathogenic mutation (*BRCA1* Q1281X); no aberrant transcript observed by cDNA analysis)
10	1022–5insT	Noncoding	N	1	H, brovca	/	/	UV/P (no aberrant transcript observed by cDNA analysis) Claes *et al* (2003)
10^b^	1441G>A	G405R	N	1	H, brca only	Y	Y/N/Y/N	UV
10	1571G>A	R448H	Y	1	F, brca only	N	N/N/N/N	UV
10	1613A>G	E462G	Y	1	F, brca only	Y	Y/Y/Y/Y	UV
11	3199A>G	N991D	Y	2	H, brca-only F, brovca	Y	N/N/N/N	UV/P (in one of the families *BRCA1* 5382insC was segregating and the patient was homozygous for N991D)
11	7052A>G	E2275G	N	1	F, brca only, male brca	Y	Y/N/N/Y	UV/P (variant cosegregating with *BRCA2* 9132delC in one of the families)
14	7285G>C	G2353R	Y	1	F, brovca	Y	Y/N/N/N	UV
14	7641A>G	T2471T	N	1	H, brca only	/	/	UV (identified in two sisters with brca)
15	7691G>A	R2488K	Y	2	H, brca only	N	Y/Y/Y/Y	UV/P (in one family not segregating with the disease; variant in linkage disequilibrium with 2166C>T (S646S))
15	7830G>C	A2534A	N	1	Sporadic	/	/	UV/P
17	8172C>T	S2648S	N	1	Sporadic	/	/	UV/P
18	8395C>G	D2723H	Y	1	H, brca only	N (negatively charged → positively charged)	Y/Y/Y/Y	UV
18	8471G>A	G2748D	Y	1	F, brca only;	Y	Y/Y/Y/Y	UV
20	8795A>C	E2856A	Y	3	F, brca-only; H, brca-only sporadic	Y	Y/Y/Y/Y	UV (not segregating with the disease in one of the families)
22	9078G>T	K2950N	Y	1	F, brca only	N (positively charged → uncharged polar)	Y/Y/Y/Y	UV (identified in two sisters with brca, one of them carrying a truncating *BRCA1* mutation)
23	9266C>T	T3013I	Y	1	F, brca only	Y	N/N/N/Y	UV
25	9520T>C	Y3098H	Y	1	F, brca only	N (positively charged → uncharged polar)	N/N/N/Y	UV
25	IVS25+9A>C	Noncoding	Y	1	H, brovca	/	/	UV/P (identified in patient carrying a *BRCA1* mutation; no aberrant transcript observed at cDNA level) Claes *et al* (2003)
27	10338G>A	R3370R	Y	1	H, brca only	/	/	UV/P (identified in a patient carrying *BRCA2* Y42C)
27	10462A>G	I3412V	Y	1	F, brca only	N	N/N/N/N	UV/P (not segregating with the disease in the family)

H=hereditary breast cancer; F=familial breast cancer; brca=breast cancer; ovca=ovarian cancer; Brca-only=site-specific breast cancer family; brovca=breast cancer family with at least one case of ovarian cancer; UV=unclassified variant; P=polymorphism; Y=yes; N=no.

a In this family two UVs were identified (*BRCA1* S1040N and D693N) and one pathogenic mutation (*BRCA2* 1617–1618delAG).

b Two genetic variants identified in the same patient.

).

When feasible, we investigated multiple affected family members in order to exclude the presence of a phenocopy. In total, 451 individuals from 349 families were selected for genetic testing (i.e. on average 1.29 individuals per family). For most patients, clinical files and pathological records were retrieved and re-evaluated. In 22 families, no affected relative was available for testing for various reasons (11 breast cancer-only families (10 FBOC and one HBOC) and 11 breast–ovarian cancer families (nine FBOC and two HBOC)), and in these families, 28 asymptomatic first-degree relatives of breast/ovarian cancer patients were analysed. Furthermore, two asymptomatic women were analysed whose mothers had died of a breast cancer diagnosed before the age of 35 years, but without a further family history.

### Mutation detection

We screened the complete coding region of *BRCA1* and *BRCA2* in all families. For the first 85 families, the following strategy was used: in the first instance, *BRCA1* exon 11 and *BRCA2* exons 10 and 11 were analysed with the protein truncation test (PTT) (Hogervorst *et al*, 1995; Friedman *et al*, 1997). If no mutation was found, all other exons and splice sites of *BRCA1* were investigated by heteroduplex analysis (HA) as described (Claes *et al*, 1999a). In 68 families in whom no mutations were identified, HA for all remaining coding exons of *BRCA2* was performed.

For 203 families, we applied another strategy. *BRCA1* exon 11 and *BRCA2* exon 11 were investigated by PTT and all remaining coding exons and splice sites with denaturing gradient gel electrophoresis (DGGE) (van der Hout *et al*, 1999), a more sensitive technique than HA. The PCR set-up was automated using a robot (RoboAmp 4200, MWG Biotech, Ebersberg, Germany).

As PTT only allows the detection of truncating mutations, direct sequencing of *BRCA1* exon 11 and *BRCA2* exon 11 was performed for the last 62 families using the dye terminator chemistry on the ABI3100. All remaining coding exons and splice sites were investigated with DGGE.

When a mobility shift was observed on HA, PTT or DGGE gels, cycle sequencing was performed using dye primer chemistry on the ALF Express (Amersham Biosciences, Buckinghamshire, England) or dye terminator chemistry on the ABI377 or ABI3100 (Applied Biosystems, Foster City, USA) according to the manufacturer's instructions.

Furthermore, a specifically designed PCR assay was used to screen all families for four recurrent *BRCA1* rearrangements (deletion exon 22, deletion exon 13, duplication of exon 13, deletion exons 8–9) (Petrij-Bosch *et al*, 1997; Puget *et al*, 1999; Rohlfs *et al*, 2000). Recently, multiplex ligation-dependent probe amplification (MLPA), a sensitive and comprehensive high-throughput test to detect single or multiple exon deletions and amplifications in the *BRCA1* gene, has been made commercially available by MRC-Holland. We analysed with MLPA 117 families in whom no mutation was identified by conventional PCR-based techniques (all 55 remaining HBOC, 52 FBOC and 10 sporadic patients).

All mutations were reconfirmed on a second blood sample from the index case before offering counselling and access to genetic testing for at-risk adult family members.

### Statistical analysis

The SPSS version 11.01 statistical analysis program was used for all analyses. The analyses were used to describe the specific characteristics (phenotypes) of the families. Characteristics included the following: at least one family member diagnosed with ovarian cancer, with bilateral breast cancer/multiple ipsilateral primary breast cancers, with male breast cancer or with both breast and ovarian cancer. These cancer diagnosis descriptors were analysed as dichotomous variables (presence *vs* absence) with the two-sided *χ*^2^ test and Fisher's exact test. Independent samples *T*-test was used to evaluate continuous variables, such as the mean age at diagnosis of breast cancer and ovarian cancer and average number of breast/ovarian cancers, between families carrying each genotype (*BRCA1*, *BRCA2*, either mutation, neither mutation). All statistical tests were two-sided. *P*-values less than 0.05 were considered to be statistically significant.

## RESULTS

### *BRCA1* and *BRCA2* mutations

**Figure 1:**
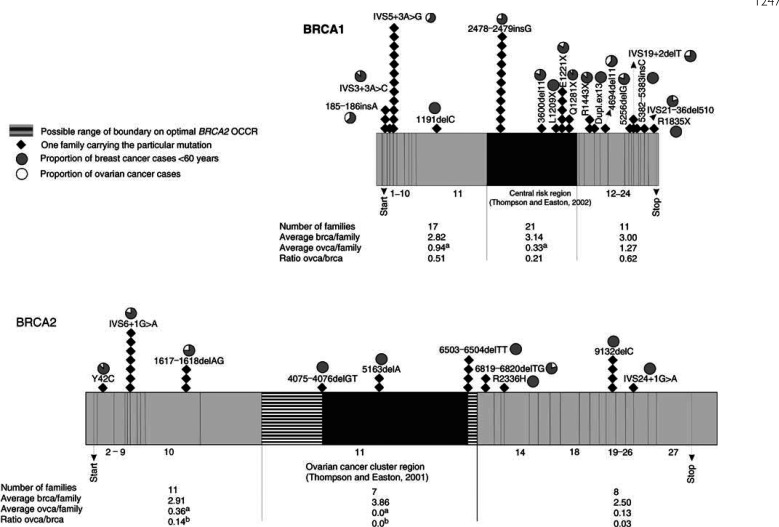
Diagrammatic representation of *BRCA1* and *BRCA2* exons, showing 49 *BRCA1* and 26 *BRCA2* germline mutations identified in Belgian breast/ovarian cancer families selected for genetic testing. A correlation with the phenotype in each mutation-positive family was made based on the ratio of ovarian cancer cases to breast cancer cases. The central regions in both genes, proposed to be associated with an increased ovarian : breast cancer ratio by Thompson and Easton (2001, 2002), are marked. Furthermore, the distribution of breast (brca) and ovarian cancers (ovca) in families according to the site of *BRCA1* and *BRCA2* mutations are indicated. (^a^) Differences in risk of ovarian cancer were statistically significant for *BRCA1* 5′ *vs* central portion (*P*=0.027) and *BRCA2* 5′ *vs* central portion (*P*=0.038). (^b^) Differences in relative risks of ovarian *vs* breast cancer were statistically significant for *BRCA2* 5′ *vs* central portion (*P*=0.017).

In total, 49 (18 distinct) *BRCA1* mutations and 26 (10 distinct) *BRCA2* mutations were identified (Figure 1

). The vast majority of the mutations are predicted to lead to a premature stop codon (39 frameshifts, 15 nonsense mutations and 24 splice site disruptions). Nontruncating amino-acid substitutions occurring in only a limited number of families were considered as unclassified variants (Table 1). For the statistical analyses, these families were considered as *BRCA1/2* mutation negative. Only *BRCA2* Y42C was thought to be a pathogenic mutation. Y42 is a highly conserved amino acid and Y to C is a radical amino-acid change, compromising *in vivo* the interaction between BRCA2 and replication protein A (Wong *et al*, 2003).

**Figure 2:**
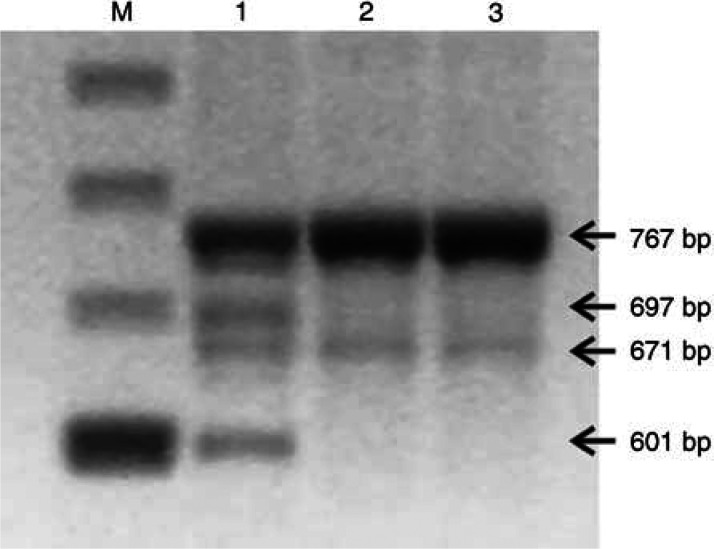
RT–PCR was performed with primers spanning exons 11–15 of the *BRCA2* gene (nucleotides 6948–7714; GenBank accession number NM_000059) on RNA extracted from lymphocytes as described by Claes *et al* (2003). RT–PCR on RNA from the patient carrying *BRCA2* R2336H (lane 1) showed a full-length fragment of 767 bp and three faster migrating bands (697 bp: skipping of *out of frame* exon 13 (stop 2345); 671 bp: skipping of *in-frame* exon 12 (deletion of 32 amino acids); 601 bp: skipping of *out of frame* exons 12 and 13 (stop 2311)). In healthy control persons (lanes 2 and 3), besides the full-length fragment, a band (671 bp) representing the skipping of *in-frame* exon 12, was also observed. M, marker.

All splice site mutations were studied at the RNA level (Claes *et al*, 2002; Claes *et al*, 2003). RT–PCR analysis for *BRCA2* R2336H in the last codon of exon 13 was not yet described and revealed the wild-type allele and three smaller transcripts, representing a complete loss of exon 13, loss of exon 12 and loss of exons 12 and 13. Loss of exon 12 was also observed in transcripts from normal individuals (Figure 2

).

The large majority of the mutations (>80%) were recurrent. *BRCA1* IVS5+3A>G and *BRCA2* IVS6+1G>A, respectively, represent the most frequent mutations in *BRCA1* and *BRCA2*. *BRCA1* IVS5+3A>G is a Belgian founder mutation (Claes *et al*, 1999b). For *BRCA2* IVS6+1G>A haplotypes could not be established since only one carrier per family was available for study. A founder effect is very likely since this mutation has not yet been reported in other populations, and the donor site of *BRCA2* exon 6 does not represent a mutational hotspot region given the limited number of mutations reported in the BIC database in this region. The recurrence of the other mutations was also due to founder effects (data not shown).

In some populations, large intragenic deletions/duplications constitute a substantial fraction of mutations (Hogervorst *et al*, 2003; Montagna *et al*, 2003). We investigated the prevalence of single or multi-exon deletions/duplications in our Belgian study population. Therefore, we analysed with MLPA 117 families in whom no mutation was identified by conventional PCR-based techniques (all 55 remaining uninformative HBOC families, 52 FBOC families and 10 sporadic patients). In none of them a genomic rearrangement was detected.

### Clinical characteristics indicative of a germline mutation

In patients with a family history of breast/ovarian cancer, significantly more mutations were identified compared to sporadic patients (71 mutations in 291 unrelated families (24.4%) and four mutations in 58 sporadic patients (6.9%); *P*=0.003). In breast–ovarian cancer families, mutation frequencies were significantly higher than in breast cancer-only families: 44.8% (30/67) *vs* 18.3% (41/224) (*P*<0.0001) (Table 2

**Table 2:** Overview of all HBOC and FBOC families investigated and mutation detection frequencies

		**FBOC**				**HBOC**			
			**#mutations found (%)**				**#mutations found (%)**		
**Phenotypes**		***N***	**BRCA1**	**BRCA2**	**BRCA**	***N***	**BRCA1**	**BRCA2**	**BRCA**
Breast–ovarian cancer	No male breast cancer patient	39	9 (23.1%)	0	9 (23.1%)	25	13 (52.0%)	6 (24.0%)	19 (76.0%)
families	At least one male breast cancer patient	0	0	0	0	3	0	2 (66.7%)	2 (66.7%)

	Total	39	9 (23.1%)	0	9 (23.1%)	28	13 (46.4%)	8 (28.6%)	21 (75.0%)

Breast cancer-only	No male breast cancer patient	155	13 (8.4%)	11 (7.1%)	24 (15.5%)	60	11 (18.3%)	3 (5.0%)	14 (23.3%)
families	At least one male breast cancer patient	6	0	2 (33.0%)	2 (33.0%)	3	0	1 (33.3%)	1 (33.0%)

	Total	161	13 (8.1%)	13 (8.1%)	26 (16.1%)	63	11 (17.5%)	4 (6.3%)	15 (23.8%)

*Total*		*200*	*22* (*11.0*%)	*13* (*6.5*%)	*35* (*17.5*%)	*91*	*24* (*26.4*%)	*12* (*13.2*%)	*36* (*39.6*%)

FBOC=familial breast and/or ovarian cancer; HBOC=hereditary breast and/or ovarian cancer.

). The highest mutation detection ratio was obtained in breast–ovarian cancer families fulfilling the criteria for hereditary disease (75%=21/28), decreasing to 23.1% (9/39) in families with familial breast and ovarian cancer (*P*<0.0001). In breast cancer-only families, mutation frequencies were 23.8% (15/63) and 16.1% (26/161) in families with, respectively, hereditary and familial breast cancer (*P*=0.185). Especially, *BRCA1* mutations conferred an increased risk for ovarian cancer: on average, there were nearly three times as many ovarian cancers in *BRCA1* families than in *BRCA2* families (0.89 *vs* 0.32; *P*=0.091). The average number of breast cancer cases was comparable (3.70 in *BRCA1* and 3.72 in *BRCA2* families; *P*=0.965). The ratio of ovarian to breast cancers was on average 0.41 : 1 for *BRCA1* families and 0.07 : 1 for *BRCA2* families (*P*=0.015).

A male breast cancer case was indicative of a *BRCA2* mutation segregating in the family (*P*=0.002) (Table 1). Furthermore, a relative with multiple primary breast cancers also increased the chance for finding a *BRCA* mutation: in 26 of 66 families (39.4%) with at least one relative diagnosed with multiple primary breast cancers, a mutation was identified compared to 45 mutations in 225 families (20%) without such a phenotype (*P*=0.002).

The mean age of diagnosis (±standard deviation) of the first female breast cancer was 45.87 (±12.80) years in mutation carriers and 49.01 (±12.35) years for persons without known mutations (*P*=0.001). On average, the age of onset was younger in *BRCA1* compared to *BRCA2* families; however, differences were not statistically significant (44.82±12.39 *vs* 48.00±13.42 years; *P*=0.086). For the mean age of diagnosis of ovarian or male breast cancer, no significant differences were obtained between mutation-positive and mutation-negative families (data not shown).

In total, four mutations were identified in 58 sporadic patients fulfilling our inclusion criteria (Table 3

**Table 3:** Phenotypical features of sporadic patients investigated (*N*=58)

	**BRCA1 mutation-positive patients**		**BRCA2 mutation-positive patients**		**Mutation positive patients**	
	***N***	**%**	**N**	**%**	**N**	**%**
41 female breast cancer patients (mean Dx 33.5, median Dx 33, range 26–52)	2	4.9	1	2.4	3	7.3
Six patients with multiple primary breast cancers (bilateral or multifocal) (mean Dx first breast cancer 39.8, median Dx 39.5, range 31–50)	0	0	0	0	0	0
Five ovarian cancer patients (mean Dx 35.4, median Dx35, range 25–52)	0	0	0	0	0	0
Two patients with both primary breast and ovarian cancer (mean Dx breast cancer 52.5 (range 50–55); mean Dx ovarian cancer 50 (range 47–53)	1	50	0	0	1	50
Four male breast cancer patients (mean Dx 49.3, median Dx 58.5, range 12–68)	0	0	0	0	0	0

58 sporadic patients	3	5.2	1	1.7	4	6.9

Dx=age at diagnosis (years).

). Three mutations (*BRCA1* IVS5+3A>G, 2626–2627delAA and *BRCA2* 6503–6504delTT) were found in 41 sporadic patients with early-onset breast cancer and one mutation (*BRCA1* E1221X) in two sporadic patients diagnosed with both breast and ovarian cancer. No mutations were found in six sporadic patients with multiple primary breast cancers, neither in five sporadic ovarian cancer patients with early-onset disease nor in four sporadic males with breast cancer.

### Variation in cancer risk by mutation position

In our series of families, we found an increased ovarian to breast cancer ratio in the 5′-end of both genes. Figure 1 suggests that, for mutations 5′ of *BRCA1* exon 11, this may result from both an increase in ovarian cancer risk and a reduction in breast cancer risk. Differences in breast cancer risk were not statistically significant. Also, for mutations in the 5′ region of *BRCA2*, a statistically significant increased incidence of ovarian cancer was observed relative to the central portion of the gene. However, our observations are based on a limited number of distinct mutations. For *BRCA2*, an ‘Ovarian Cancer Cluster Region’ (OCCR) in the middle third of the gene was proposed by Gayther *et al* (1997). Surprisingly, in our study no ovarian cancer cases were recorded in seven families bearing three distinct mutations in the *BRCA2* OCCR.

## DISCUSSION

We identified 75 disease-causing mutations in 349 Belgian breast/ovarian cancer families selected for genetic testing. The prevalence of *BRCA1* mutations was approximately twice the prevalence of *BRCA2* mutations. Most strikingly, six mutations (*BRCA1* IVS5+3A>G, 2478–2479insG, E1221X, and *BRCA2* IVS6+1G>A, 6503–6504delTT and 9132delC) accounted for nearly 60% of all mutations identified. *BRCA1* 2478–2479insG and *BRCA2* IVS6+1G>A have not yet been reported in other populations. *BRCA1* IVS5+3A>G is a Belgian founder mutation that has also been found in a few German, Dutch and French families (Claes *et al*, in preparation). *BRCA1* E1221X and *BRCA2* 6503–6504delTT & 9132delC have been reported in several populations worldwide (BIC database). Given the proportion of all breast/ovarian cancer families in our population attributable to recurring mutations, a cost-effective stepwise molecular screening strategy of *BRCA1* and *BRCA2* may be applied in the future. A first-stage analysis, covering the recurrent mutations, can be offered to a substantial number of families, then, if a negative test result is obtained, more stringent risk criteria can be applied for complete analysis of the genes. Further examples of founder mutations in particular regions of Belgium may be found; the majority of the patients we analysed are living in North-West Belgium. Therefore, larger studies are required.

Despite the fact that Belgians historically have been in contact with many populations, only a limited number of founder mutations from other countries were detected. *BRCA1* 5382insC, an Ashkenazi Jewish founder mutation and the most frequent mutation in many European populations, was identified in only one Belgian family. Besides *BRCA1* 5382insC, no other Jewish mutations were detected in our patient population. The deletion of *BRCA1* exon 22, the most recurrent Dutch mutation, was identified in one Belgian patient with a Dutch mother. The *BRCA1* exon 13 6-kb duplication, a mutation likely to be derived from a British ancestor (The BRCA1 Exon 13 Duplication Screening group, 2000), was found once. Besides these two rearrangements, no other mutations involving one or more exons were detected with MLPA in all 55 remaining uninformative HBOC families, in 52 FBOC families and 10 sporadic patients in whom no mutations were identified with conventional PCR-based techniques. These preliminary data suggest that genomic rearrangements in *BRCA1* do not have a major contribution in Belgian breast/ovarian cancer families. However, as indicated before, we mainly screened patients living in North-West Belgium; in other regions, this kind of mutations may be more prevalent.

Besides true pathogenic mutations, we identified several ‘unclassified variants’. Many of them were identified in high-risk families and occurred at amino acids that display substantial evolutionary conservation. Some of them were likely to be polymorphic for various reasons (Table 1). Most of the unclassified variants in our study were infrequent in our population and not reported before. A very high number of properly selected control individuals would be needed to search for statistically significant associations of these alleles with breast/ovarian cancer. We recently started LOH analysis for several variants to test the presumed association with the *BRCA* loci. We are also investigating if some of the unclassified variants affect correct splicing by disrupting functional exonic splicing enhancer sequences, as described for a *BRCA2* amino-acid substitution (Fackenthal *et al*, 2002).

Our study permits to estimate the prevalence of *BRCA1* and *BRCA2* mutations in a Belgian patient population referred to a family cancer clinic. In 24.4% of the patients with a family history of breast/ovarian cancer, a mutation was identified. In families with at least one relative with ovarian cancer (*P*<0.0001), multiple primary breast cancers (*P*=0.002) or male breast cancer (*P*=0.002) significantly more mutations were identified compared to families without such phenotypes (Table 2). The literature is not unanimous about the predictive value of multiple primary (ipsilateral or contralateral) breast cancers for finding a mutation. Our results are in agreement with the studies of Bergthorsson *et al* (2001), de la Hoya *et al* (2002) and Ford *et al* (1998); however, others failed to demonstrate such a predictive value (Couch *et al*, 1997; Steinmann *et al*, 2001). Male breast cancer in combination with a family history of breast/ovarian cancer was indicative of finding a *BRCA2* mutation (*P*=0.002), which is consistent with a recent population-based British study (Basham *et al*, 2002). In none of the four Belgian sporadic male breast cancer patients a mutation was identified. However, *BRCA2* mutation prevalence in sporadic male breast cancer has been found to be as high as 33 and 21% in the Hungarian and Swedish population (Haraldsson *et al*, 1998; Csokay *et al*, 1999). Larger studies in Belgian patients are required before definite conclusions about mutation frequencies in this group can be drawn.

In mutation-negative families, the average age of onset of female breast cancer, but not male breast cancer, tended to be higher than in families in whom a mutation was identified. The mean age of onset for ovarian cancer was comparable in mutation-positive and mutation-negative families. These observations are in agreement with large studies on ovarian cancer patients (Risch *et al*, 2001; Frank *et al*, 2002). It has repeatedly been shown that only a small percentage of *BRCA*-positive ovarian cancer cases occur at ages <40 years (Boyd *et al*, 2000; Liede *et al*, 2002).

As early onset breast/ovarian cancer and the occurrence of multiple primary breast and/ovarian cancers in a single individual adds considerably to the prior probability of a mutation being present, we evaluated the prevalence of *BRCA1/2* mutations in patients with these clinical characteristics and no further family history. Three recurrent mutations (*BRCA1* E1221X & IVS5+3A>G and *BRCA2* 6503–6504delTT) and one novel mutation (*BRCA1* 2626–2627delAA) in 54 such patients were identified. For the recurrent mutations, a *de novo* event was highly unlikely. Haplotype analysis had revealed identical alleles with other Belgian patients carrying *BRCA1* E1221X and IVS5+3A>G, respectively (data not shown). The patient with the *BRCA2* 6503–6504delTT mutation was bearing in addition two downstream polymorphisms (IVS24-16T>C and K3326X) that had been observed in all our other patients carrying the *BRCA2* 6503–6504delTT mutation. *BRCA1* 2626–2627delAA has not yet been reported in the BIC database and was identified in a sporadic patient diagnosed with breast cancer at the age of 27 years. A *de novo* event could not be ruled out as DNA of the parents was not (yet) available.

In only 7.5% of the sporadic patients diagnosed with breast cancer at young age, *BRCA1/2* mutations were identified. We hypothesise a possible role for genetic variants in DNA double-strand break repair genes in this patient group, since a significant proportion of these patients showed elevated chromosomal radiosensitivity by *in vitro* assays (Baeyens *et al*, 2002).

In hereditary *breast cancer-only* families, mutation detection ratios were low (23.8%) compared to hereditary *breast–ovarian cancer* families (75%) (*P*<0.0001). Mutation frequencies did not statistically significantly differ between breast cancer-only families with and without an autosomal dominant inheritance pattern of the disease (23.8 *vs* 16.1%; *P*=0.085). As breast cancer is relatively frequent in Belgium, familial clustering of breast cancer may have occurred by chance in some families. In a few families, mutations undetectable by the techniques used may be present (e.g. regulatory mutations) or some of the genetic variants reported as being of ‘uncertain significance’ may be characterised as deleterious in the future. Nevertheless, our findings strongly suggest a role for additional breast cancer susceptibility genes.

From a clinical perspective, it would be most interesting to gain an insight into a possible relationship between mutation site and relative risk of breast or ovarian cancer. We found that in families with mutations occurring 5′ of *BRCA1* exon 11 on average a significantly higher number of ovarian cancers were present than in families with mutations in the central portion of the gene (Figure 1). This trend is not consistent with the study of The Breast Cancer Linkage Consortium (BCLC) (Thompson and Easton, 2002) reporting an increased ovarian to breast cancer ratio in the central region of *BRCA1* due to a lower breast cancer risk. Furthermore, they found a reduced ovarian cancer risk associated with mutations in the 3′ part of the gene, a trend that was not observed in our study.

For *BRCA2,* the BCLC study (Thompson and Easton, 2001) revealed an increased ovarian to breast cancer ratio for OCCR mutations, due to a reduced absolute risk of breast cancer. This observation was not confirmed in our study population. In none of our families bearing a *BRCA2* OCCR mutation ovarian cancer was part of the phenotypes. Several other investigators (for instance, Frank *et al*, 1998; Ikeda *et al*, 2001; de la Hoya *et al*, 2002) also failed to demonstrate an increased incidence of ovarian cancer in the *BRCA2* OCCR. In our study, mutations occurring 5′ of the OCCR were significantly associated with a higher ovarian cancer risk relative to the central portion of the gene.

As the data from different studies are not consistent, we think that associations between mutation position and phenotype are not sufficiently strong to influence genetic counselling and management of individual families. It is well known that large variations in cancer risks are also observed in families bearing the same mutation, suggesting the involvement of genetic and/or environmental modifiers. For counselling of affected families, it may be wiser to take into account the previous history of the family.
